# Evaluation of a portable multiplex real-time pcr assay for the detection of monkeypox virus and non-variola orthopoxviruses

**DOI:** 10.1186/s12985-026-03195-1

**Published:** 2026-06-22

**Authors:** Shanshan Li, Nabojeet Das, Natalie Miller, Ariel Caudle, Kimberly Wilkins, Theodora Khan, Stefan Pfotenhauer, Amy Saunders, Christina L. Hutson, Kodumudi S. Venkateswaran, William M. Nelson, Neeraja Venkateswaran, Panayampalli S. Satheshkumar, Michael B. Townsend

**Affiliations:** 1 Poxvirus and Rabies Branch, Centers for Disease Control and Prevention, Atlanta, GA USA; 2 Tetracore Inc., Rockville, MD USA

**Keywords:** Mpox, Monkeypox virus, PCR, Point-of-care

## Abstract

Monkeypox (mpox) is the most significant orthopoxvirus (OPXV) infection in humans since smallpox eradication. The World Health Organization (WHO) has twice declared public health emergency of international concern due to the expansive spread of clade I and II mpox cases, thus prompting public health scientists to develop rapid and effective diagnostic tools. Clinical presentations of monkeypox can overlap with other vesiculopustular diseases, particularly those caused by Varicella-Zoster virus (VZV), complicating accurate diagnosis in decentralized or resource-limited settings. In this study, we performed an in-lab evaluation of a multiplexed real-time PCR on a portable platform (T-COR 8) for the detection of monkeypox virus (MPXV) and non-variola orthopoxvirus (NVAR-OPXV), and differentiation from varicella-zoster virus (VZV). The T-COR 8 platform required minimal technical skill, limited hands-on time, and generated results for 8 samples within 1 h using crude specimens, without the need for nucleic acid extraction. The performance characteristics were investigated using MPXV and VZV clinical sample remainders and a panel of laboratory cultured viruses. Testing of 116 clinical remainder specimens in the MPXV/NVAR-OPXV assay yielded 100% sensitivity, 97.8% specificity, and 99.1% agreement with prior test results. The MPXV/NVAR-OPXV/VZV assay detected and correctly differentiated MPXV and VZV in a limited set of clinical samples (*n* = 39). The MPXV assay demonstrates specificity for MPXV, except for Akhmeta virus (AKMV), out of the 20 different species/isolates tested, including 15 orthopoxviruses and 4 non-orthopoxviruses, and VZV. These findings support the T-COR 8 platform as a rapid, accurate, and field-deployable diagnostic tool suitable for expanding monkeypox testing capacity.

**Supplementary Information:**

The online version contains supplementary material available at https://doi.org/10.1186/s12985-026-03195-1.

## Background

Following the successful eradication of smallpox, monkeypox (mpox) has emerged as the most significant orthopoxvirus (OPXV) infection in humans. Monkeypox virus (MPXV), is a member of the orthopoxvirus genus that contains variola, cowpox, and vaccinia viruses, with vaccinia viruses primarily used for vaccinations in humans. Historically, mpox was considered a zoonotic disease endemic to regions of Central and West Africa, with limited human-to-human transmission. However, recent outbreaks have demonstrated sustained transmission and widespread geographic expansion, including multi-country epidemics involving both clade I and clade II viruses [1, 2]. These developments prompted the World Health Organization (WHO) to declare mpox a Public Health Emergency of International Concern (PHEIC) on two occasions, highlighting the need for improved global diagnostic capacity and outbreak response [3].

Since January 2022, a total of 66,870 mpox cases in 34 member states across Africa have been laboratory confirmed, with 289 death [3, 4]. Further, in 2023 alone, DRC reported approximately 60,967 suspected cases, but only 7,438 cases (12.2%) were confirmed by lab testing [5]. Thus, there is a pressing need for additional testing resources. Detection via clinical presentation is possible but challenging, as there are other blistering diseases like Herpes, Varicella-Zoster virus (VZV) and measles which have historically accounted for up to 50% of suspected mpox cases in endemic areas [6]. Options for rapid antigen or lateral flow-based point-of care test have been developed but are of limited use given low sensitivity [7], and none are recommended by the World Health Organization [8]. There have also been numerous efforts to develop LAMP-based assays for mpox; few of these have been commercialized or remain readily available [9–11]. Currently, real-time PCR remains the gold standard for detection of MPXV. Real-time PCR is highly sensitive but utilizes lab equipment that is bulky, requires a continuous power-source, and require well-trained laboratorians to perform the test [12]. Additionally, conventional PCR workflows typically require nucleic acid extraction.

**Fig. 1:**
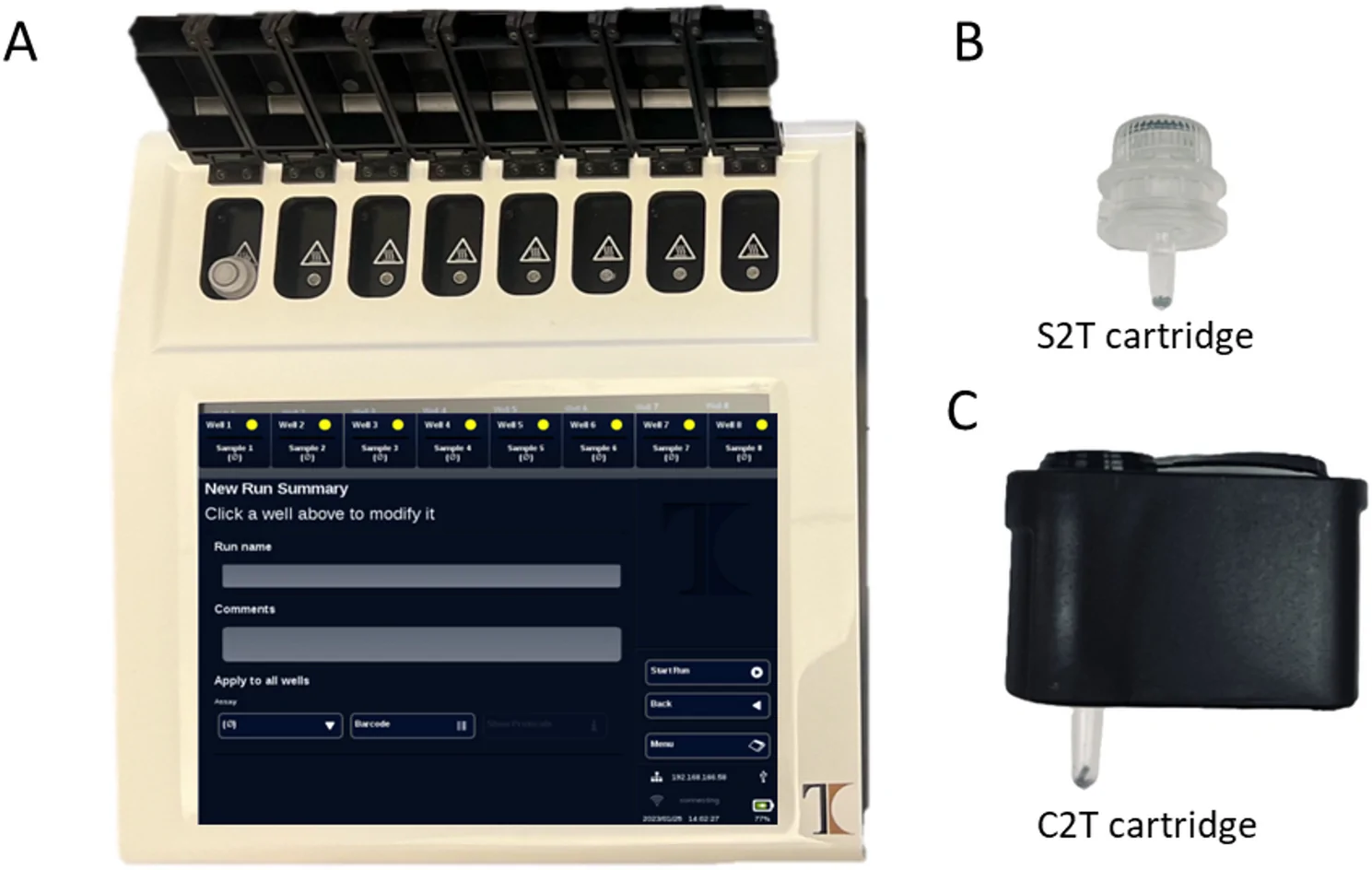
(**A**) T-COR 8 real-time PCR Thermocycler Instrument and layout showing application window. The S2T cartridge (**B**) or C2T cartridge (**C**) used per assay. The T-COR 8 thermocycler weighs 9.7 lb (4.4 kg), has dimensions of 11.9 × 10.9 × 4.25 in (30.2 × 27.7 × 10.8 cm), and provides a battery life of approximately 2 h during active thermocycling

To address these limitations, there is increasing interest in portable and simplified molecular diagnostic platforms that can deliver rapid and reliable results with minimal sample processing. Multiplex PCR assays capable of simultaneously detecting MPXV and differentiating it from clinically similar pathogens such as VZV offer additional advantages in improving diagnostic accuracy and efficiency [13]. Several portable PCR platforms including Biomeme Franklin and Hirsi bCUBE, have been developed in recent years for point-of-care use [14, 15]. These systems can provide rapid results, simplified workflows, and reduced reliance on centralized testing as the T-COR 8 does, but neither currently offers MPXV detection. In this study, we utilized the portable T-COR 8 real-time PCR thermocycler Platform (Tetracore, Inc., USA; Fig. 1) in a lab-based evaluation using two different multiplex assays, MPXV/NVAR-OPXV and MPXV/NVAR-OPXV/VZV, for the detection and differentiation of MPXV from other poxviruses and VZV using clinical reminder samples and cultured viruses. The T-COR 8 real-time PCR thermocycler platform is a compact, portable, battery-operated device that requires no extraction and features an all-in-one cartridge. It weighs 9.7 lb (4.4 kg), has dimensions of 11.9 × 10.9 × 4.25 in (30.2 × 27.7 × 10.8 cm), and provides a battery life of approximately 2 h during active thermocycling. Results were compared to prior findings using traditional laboratory PCR or real-time PCR assays [16].

## Methods

### MPXV and VZV clinical specimens

MPXV clinical remainder specimens utilized in this study were received at the Centers for Disease Control and Prevention (CDC) for MPXV-specific testing during the 2022 outbreak in the United States ( Table 1). The MPXV Clade II real-time PCR test serves as laboratory comparator results, representing original diagnostic results, and samples were not re-tested in parallel with the T-Cor 8 assay as part of this study. The MPXV clinical specimens were received as dry lesion swabs from suspect mpox cases. Swabs were eluted into 1X PBS for clinical testing; remainder eluate was stored frozen until testing herein with the T-COR platform. A convenience sampling of 116 clinical remainder specimens including those with a range of prior Ct results (Ct range = 20–40) were identified and tested using MPXV/NVAR-OPXV assay on the portable T-COR 8 thermocycler. Of these, 88 specimens were included in a pilot study to assess assay suitability. Following this initial work, an additional 28 specimens were identified and used in a blind study to ensure elimination of bias by testers. To assess impact of disease progression or patient age on test results, sampling time points were aligned according to days post-rash onset (DPR) and categorized into four groups: Days 0–5, Days 6–10, Days 11+, and unknown onset. Patient ages varied and were stratified into five age groups: <18 years, 18–29 years, 30–39 years, 40–49 years, and ≥ 50 years. Assessment of the MPXV/NVO-OPXV/VZV assay was performed by a convenience selection of 20 MPXV specimens (Ct range 20–40), and by request of 20 VZV clinical remainder specimens obtained from the CDC Viral Vaccine Preventable Diseases Branch. A total of 19 VZV specimens (9 VZV positive and 10 VZV negative) were identified from prior surveillance projects. All samples, including MPXV and VZV specimens, were deidentified prior to testing, and all testing, inclusive of original clinical testing and TCOR-8 testing, was performed at CDC laboratories. Use of de-identified clinical remainders in this study was reviewed by CDC, deemed research not involving human subjects, and was conducted consistently with applicable federal law and CDC policy. §^1^

**Table 1:** Patient demographics for pilot study and blind study

		Pilot study (*n* = 88)	Blind study (*n* = 28)	Total (*n* = 116)
Sex	Female	10/88 (11.4%)	3/28 (10.7%)	13/116 (11.2%)
	Male	31/88 (35.3%)	25/28 (89.3%)	56/116 (48.3%)
	N/A*	47/88 (53.4%)	0/28 (0%)	47/116 (40.5)
Age	< 18	2/88 (2.3%)	0/28 (0%)	2/116 (1.7%)
	18–29	20/88 (22.7%)	6/28 (21.4%)	26/116 (22.4%)
	30–39	32/88 (36.3%)	9/28 (32.1%0	41/116 (35.3%)
	40–49	20/88 (22.7%)	6/28 (21.4%)	26/116 (22.4%)
	50+	14/88 (15.9%)	7/28 (25.0%)	21/116 (18.1%)
Days post rash onset	0–5	14/88 (15.9%)	5/28 (17.9%)	19/116 (16.4%)
	6–10	4/88 (4.5%)	6/28 (21.4%)	10/116 (8.6%)
	11+	6/88 (6.8%)	7/28 (25.0%)	13/116 (11.2%)
	N/A*	64/88 (72.7%)	10/28 (35.7%)	74/116 (63.8%)

*N/A, data not available

### Laboratory grown OPXV and VZV antigens

The panel of OPXV utilized for the specificity study were selected from isolates available in our laboratories. Isolates had previously been identified from clinical or surveillance samples, cultured in vitro in BSC40 (African Green Monkey Kidney Epithelial cells). Crude preparations used for testing were prepared by 3-times freeze/thaw of infected BSC40 cells in Dulbecco’s Modified Eagle Medium containing 2% FBS and 1% penicillin/streptomycin after 2–3 days at 37 °C. Preparations were then subjected to low speed centrifugation at 1000 RPM (300Xg) and supernatant collected to remove cellular debris. The VZV II antigen (Catalog #7740, Lot #M30007017, Meridian Life Science, Inc, Memphis, TN) was derived from the Ellen viral strain and was prepared from cultured human fibroblast cells. Testing of inactivated variola virus (VARV) was approved by the WHO Advisory Committee on Variola Virus (ACVVR). Isolates are identified in Supplemental Table 1.

### Prior clinical results testing and specimen assignment

Clinical remainder MPXV specimens had been tested with the CDC MPXV Clade II real-time PCR test (formerly named the West Africa (WA) MPXV test) using a previously reported protocol [17]. Briefly, swabs were resuspended in 500 uL of PBS and eluted. A total of 100 ul of eluate was used for DNA extraction using the Qiagen EZ1robot system and EZ1 DNA Tissue Kit (Qiagen, Valencia, CA) and purified DNA eluted into 50 µL, of which 2 µL was utilized for the real-time PCR test. The Ct values reported by the test were interpreted with Ct < 37 positive, Ct between 37 and 40 inconclusive, and Ct > 40 negative. VZV remainder specimens had been tested using Orf62 primers in a traditional PCR assay as previously described [18]. Specimens were provided with prior test results of positive or negative for VZV.

### T-COR 8 MPXV/NVAR-OPXV and MPXV/NVAR-OPXV/VZV assays

The T-COR 8 real-time thermocycler instrument has 8 individually addressable positions, allowing for simultaneous testing of up to 8 samples (Fig. 1A). The MPXV/NVAR-OPXV testing was conducted using the S2T cartridge (Fig. 1B), which contains all materials in a liquid state and requires storage at -20 °C. To improve its suitability for field use, Tetracore subsequently developed the C2T cartridge (Fig. 1C), which lyophilizes all materials into a powdered form, allowing for room temperature storage and enhanced stability. The MPXV/NVAR-OPXV/VZV testing was conducted using the C2T cartridge using manufacturer’s protocol (Tetracore, Inc, Rockville, MD). Briefly, clinical specimens were diluted 1:4 in the provided sample dilution buffer and 50 µL applied to S2T or C2T cartridge. After sample loading, it is briefly centrifuged to mix with the PCR reaction mix, and the cartridge loaded into the thermocycler. Laboratory grown isolates and VZV antigen were heat inactivated at 56 °C for 1 h prior to testing, and then were diluted 1:100 in sample dilution buffer and applied to the C2T cartridge. The T-COR 8 PCR assays consists of 40 cycles of 98 °C for 10-seconds and 60 °C for 15-seconds and have a Ct detection cut-off of 38.

In the MPXV/NVAR-OPXV assay, MPXV was detected using MPXV generic primers [17] in FAM channel, and NVAR-OPXV was detected using E9L primers [19] in the CFR channel. An inhibition control (IC) was detected in the DFO channel, and the endogenous control (RNase P) was detected in the Cy5 channel (Supplemental Table 2). In the MPXV/NVAR-OPXV/VZV assay, the endogenous control was removed and replaced with VZV, which was detected in the Cy5 channel. The results were reported as detected, not detected, or invalid. Specimens were tested once unless an invalid result was given, in which case the specimen was retested using the same conditions per manufacture instructions.

### Statistical analyses

Samples utilized in the clinical specimen evaluation were identified as True Positives (TP) and True Negatives (TN) based on testing prior results from CDC MPXV Clade II real-time PCR test or the VZV ORF62 PCR test. Statistics for the MPXV/NVAR-OPXV assessment were performed according to the following: Sensitivity, TP / (TP + FN); Specificity, TN / (TN + FP); Positive Predictive Value (PPV), TP / (TP + FP); and Negative Predictive Value (NPV), TN / (TN + FN). The agreement rate (%) was calculated as follows: (number of matching results from both assays / total sample number) × 100%. The 95% confidence intervals (95% CI) calculated using the exact (Clopper–Pearson) method. Statistical analyses were performed only on the pilot and blind testing using the MPXV/NVAR-OPXV assay. Due to the small sample size, statistical analyses were not conducted for clinical sample or virus isolate specificity testing using the MPXV/NVAR-OPXV/VZV assay. Changes in Ct comparisons between the prior test results (Clade II real-time PCR) and the T-COR 8 MPXV assays were analyzed using Tukey’s comparisons test with adjusted p-values reported.

## Results

### Pilot and blind evaluation of T-COR 8 MPXV/NVAR-OPXV assay with clinical samples

We analyzed a total of 88 clinical specimens in the pilot study, and an additional 28 clinical specimens in the blind study (Table 2). The clinical specimens were provided to CDC for confirmatory mpox diagnosis and tested by CDC Clade II MPXV real-time PCR test. Positives and negatives were defined based on a gold-standard test, in this case is the laboratory-based CDC Clade II MPXV real-time PCR test. The pilot study MPXV/NVAR-OPXV results identified 52 positives, 35 negatives, and 1 false positive, yielding a 98.8% positive percent agreement with the CDC Clade II MPXV real-time PCR test results (Supplemental Table 3). One sample obtained from a male participant aged 20–29 years, collected three days post-rash onset, yielded a false positive result. Upon re-test with the T-COR 8, the expected result of negative was found. The inconsistent result may have been due to sample handling error or a potential cartridge-related artifact; repeat testing from the same sample tube did not reproduce the initial finding. Additionally, three samples produced “invalid” results during initial T-COR 8 testing. Following the subsequent retest, all three samples’ results obtained aligned with those from the CDC Clade II MPXV real-time PCR test. Following this effort, 28 samples were prepared for a blind study; these included 18 confirmed positives and 10 confirmed negatives. The MPXV/NVAR-OPXV assay yielded 100% positive precent agreement compared to the CDC Clade II MPXV real-time PCR test results (Table 2). On average, the Ct difference between the initial Clade II MPXV test and T-COR 8 MPXV was less than one Ct, and differences were not significant when assessed by patient sex or age, but were different when assessed by time since rash/symptom onset. Swabs with resulting MPXV positive test results taken at 11 + days post rash (DPR) onset (*n* = 7, mean Ct∆ = -1.23) were better detected in the T-COR 8 (Supplemental Fig. 1) relative to those collected 0–5 DPR (*n* = 3, Ct∆=0.00; *p* = 0.027) or 6–10 DPR (*n* = 3, mean Ct∆=-0.2; *p* = 0.012). All pilot and blind study results are shown in Supplemental Tables 3 and Supplemental Table 4.

**Table 2:** T-COR8 test results with the MPXV/NVAR-OPXV assay

	Pilot study	Blind study	Combined total
Total specimens, *n*	88	28	116
Positives, n	52	18	70
Negatives, n	35	10	45
False positives, n	1*	0	1*
False negatives, n	0	0	0
Sensitivity (95% CI)	100% (93.2–100%)	100% (81.5–100%)	100% (94.9–100%)
Specificity (95% CI)	97.1% (85.1–99.9%)	100% (69.2–100%)	97.8% (88.4–99.9%)
Positive predictive value (PPV) (95% CI)	98.1% (89.7–100%)	100% (81.5–100%)	98.6% (92.3–100%)
Negative predictive value (NPV) (95% ci)	100% (90.0-100%)	100% (69.2–100%)	10% (92.1–100%)
Agreement (95% CI)	98.8% (93.8–100%)	100% (87.7–100%)	99.1% (95.3–100%)

95% confidence intervals (95% CI) calculated using the exact (Clopper–Pearson) method

*Upon retest, sample result was negative. This is the only specimen that underwent repeat testing

### Differentiation of MPXV and VZV using T-COR 8 MPXV/NVAR-OPXV/VZV assay with clinical specimens

A total of 20 clinical specimens suspected of MPXV infection, and 19 clinical specimens suspected of VZV infection were analyzed using the MPXV/NVAR-OPXV/VZV assay (Table 3). The MPXV specimens were previously tested by CDC Clade II MPXV real-time PCR test and included 10 positive and 10 negative specimens. T-COR 8 results were consistent with prior findings, with 10 MPXV detected and 9 of 10 NVAR-OPXV detected, and 10 negatives all not detected. Notably, one previously Clade II MPXV negative specimen was detected as VZV with the MPXV/NVAR-OPXV/VZV assay. The VZV specimens were previously tested by PCR using Orf62 primers and included 9 positive and 10 negative specimens. Results for all 19 agreed with prior VZV PCR results. No co-infections were identified among the samples tested. Overall, the results suggest MPXV/NVAR-OPXV/VZV assay can detect MPXV and differentiate from VZV in suspected clinical samples.

**Table 3:** MPXV/NVAR-OPXV/VZV assay with clinical specimens

Virus category	Sample ID	PCR results*	T-COR 8 MPXV/NVAR-OPXV/VZV assay**				
			MPXV	Inhibition control	NVAR-OPXV	VZV	Interpretation
Clinical samples of 2022 monkeypox virus (MPXV) infection	P46	23.4	26.3	28.5	27.3		Detected: MPXV, NVAR-OPXV
	P47	23.3	25.5	28.2	26.4		Detected: MPXV, NVAR-OPXV
	P48	19.7	23.3	34.3	24.2		Detected: MPXV, NVAR-OPXV
	P49	22.9	26.4	28.3	27.1		Detected: MPXV, NVAR-OPXV
	P50	24.5	27.2	28.9	28.3		Detected: MPXV, NVAR-OPXV
	P51	22.4	28.6	29.1	29.3		Detected: MPXV, NVAR-OPXV
	P52	22.9	26	28.6	26.8		Detected: MPXV, NVAR-OPXV
	B17	35.8	34.4	30.0			Detected: MPXV
	B20	30.9	32.5	29.4	33.0		Detected: MPXV, NVAR-OPXV
	B9	32.4	33.0	29.8	33.6		Detected: MPXV, NVAR-OPXV
	P56	40		32.3			Not Detected
	P57	40		29.6			Not Detected
	P58	40		29.8			Not Detected
	P59	40		30.1			Not Detected
	P60	40		30.1			Not Detected
	P61	40		29.7			Not Detected
	P62	40		29.5		23.7	Detected VZV
	P63	40		29.7			Not Detected
	P64	40		29.4			Not Detected
	P66	40		29.0			Not Detected
Clinical samples of Varicella-zoster virus infection	V1	Positive for VZV		29.7		23.3	Detected: VZV
	V2	Positive for VZV		29.6		32.4	Detected: VZV
	V3	Positive for VZV		30.2		28.4	Detected: VZV
	V4	Positive for VZV		29.6		27	Detected: VZV
	V5	Positive for VZV		30.2		32.1	Detected: VZV
	V6	Positive for VZV		29.5		24.2	Detected: VZV
	V7	Positive for VZV		30		23.5	Detected: VZV
	V8	Positive for VZV		30.1		28.9	Detected: VZV
	V9	Positive for VZV		30.1		31.1	Detected: VZV
	V10	Not Detected		29.7			Not Detected
	V11	Not Detected		29.5			Not Detected
	V12	Not Detected		29.8			Not Detected
	V13	Not Detected		30.1			Not Detected
	V14	Not Detected		29.8			Not Detected
	V15	Not Detected		29.8			Not Detected
	V16	Not Detected		29.8			Not Detected
	V17	Not Detected		29.8			Not Detected
	V18	Not Detected		29.7			Not Detected
	V19	Not Detected		29.6			Not Detected

*The MPXV specimens were tested by CDC Clade II MPXV real-time PCR test with Ct < 37 MPXV positive and ≥ 40 negative. The VZV specimens were tested by PCR using Orf62 primers

**T-COR 8 MPXV/NVAR-OPXV/VZV assay: MPXV was detected in FAM channel, inhibition control in DFO channel, NVAR-OPXV in CFR channel, and VZV in Cy5 channel

**Table 4:** Specificity of MPXV/NVAR-OPXV/VZV with lab-grown virus

Virus sample	T-COR 8 MPXV/NVAR-OPXV/VZV assay				
	MPXV	Inhibition control*	NVAR-OPXV	VZV	Interpretation
MPXV-clade IIa	20.1		20.9		Detected: MPXV, NVAR-OPXV
MPXV-clade IIb	21.7		22.1		Detected: MPXV, NVAR-OPXV
MPXV-clade Ia	17.0		18.0		Detected: MPXV, NVAR-OPXV
MPXV-clade Ib	21.0		21.7		Detected: MPXV, NVAR-OPXV
AKMV	31.4	29.5	31.4		Detected: MPXV, NVAR-OPXV
VACV-WR		27.4	23.9		Detected: NVAR-OPXV
VACV-MVA		28.3	24.8		Detected: NVAR-OPXV
ECTV		28.9	28.0		Detected: NVAR-OPXV
CPXV-FIN2000		29.6	32.1		Detected: NVAR-OPXV
CPXV-BR			23.8		Detected: NVAR-OPXV
VZV		28.9		20.2	Detected: VZV
CPXV-GER98		29.8			Not Detected
Skunkpox		29.4			Not Detected
Taterapox		29.4			Not Detected
Batpox		29.7			Not Detected
BRPV		29.0			Not Detected
Deerpox		29.8			Not Detected
Raccoonpox		29.3			Not Detected
Volepox		29.7			Not Detected
VARV-V68-258		27.4			Not Detected
BSC-40 cell lysate		29.9			Not Detected

*The Inhibition Control is not always reported if amplification is detected in a test channel

### Specificity of the T-COR 8 MPXV/NVAR-OPXV/VZV assay

**Fig. 2:**
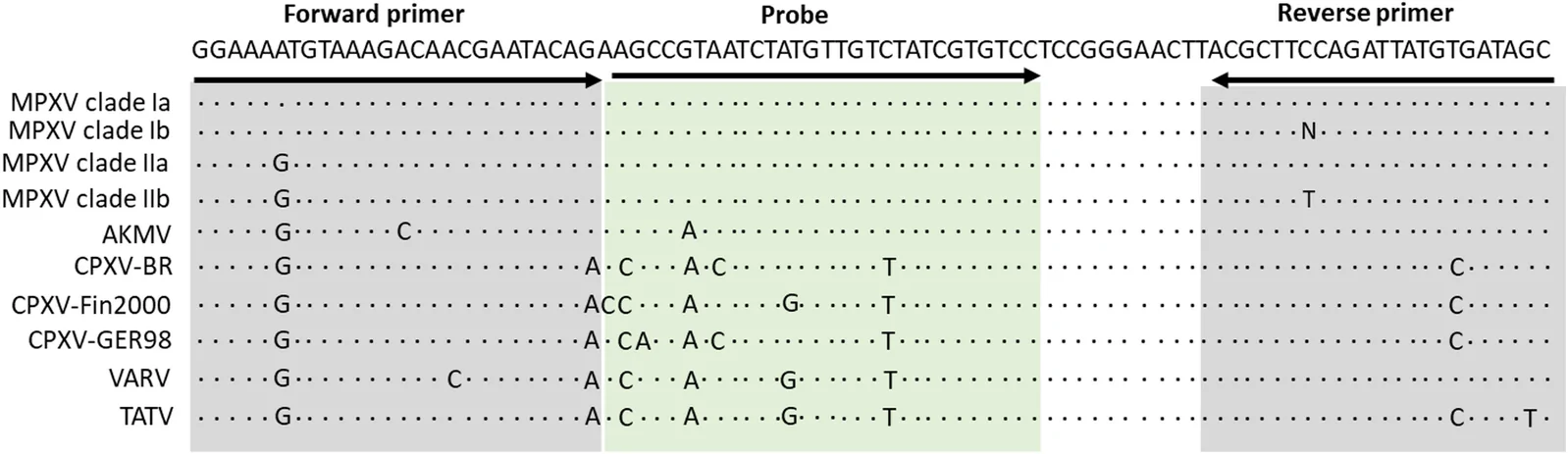
Alignment of MPXV primers and probe with poxviral DNA. The primers and probe for each are aligned with the targeted sequence of DNA within several orthopoxviral species. VACV-WR, VACV-MVA, ECVT, BRPV, Skunkpox, Raccoonpox, Volepox, deerpox, batpox, tanapox are not shown (6 + mismatches on any target sequence). Primer sequences are MPXV-F (GGAAAATGTAAAGACAACGAATACAG), Probe (AAGCCGTAATCTATGTTGTCTATCGTGTCC), and MPXV_R (GCTATCACATAATCTGGAAGCGTA)

**Fig. 3:**
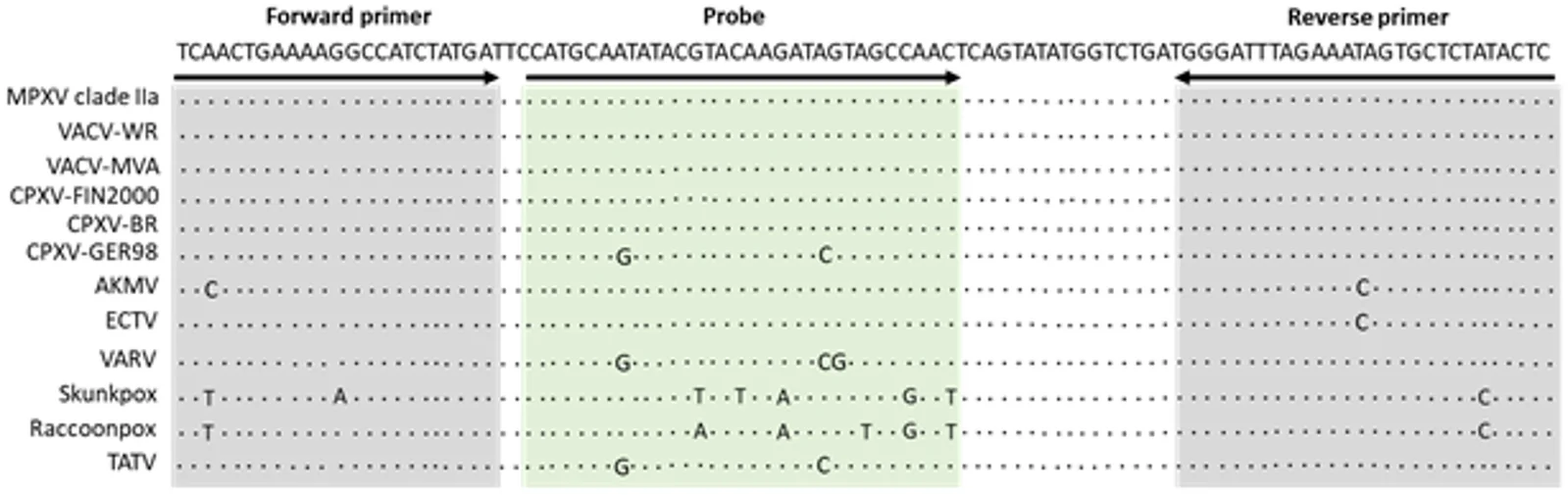
Alignment of NVAR-OPXV (E9L) primers and probe with poxviral DNA. The primers and probe for each are aligned with the targeted sequence of orthopoxviral species. E9L target gene is presented as found in the genome on the reverse strand. Volepox, deerpox, batpox, tanapox are not shown (6 + mismatches on any target sequence). MPXV clades Ia, Ib, and IIb are not shown as they share identical sequence within E9L primers and probe binding regions. Primer sequences are Forward (TCAACTGAAAAGGCCATCTATGA) Probe (CCATGCAATATACGTACAAGATAGTAGCCAAC), and Reverse (GAGTATAGAGCACTATTTCTAAATCCCA)

The specificity of the MPXV/NVAR-OPXV/VZV assay was assessed utilizing an extensive panel of 19 different poxvirus isolates, and a commercial VZV antigen/lysate (Table 4). The MPXV channel detected the MPXV clade Ia, Ib, IIa, and IIb isolates, also Akhmeta virus (AKMV). AKMV, which was discovered in 2019 and most genetically similar to cowpox viruses [14], has to date only been detected in the country of Georgia. Sequence alignment of the MPXV generic primers used in the MPXV/NVAR-OPXV/VZV assay revealed that the primer and probe sequences are matched well to MPXV genome, while two extra mismatches were identified in the corresponding regions of the AKMV genome. One is within the MPXV forward primer binding site and one is within the probe binding region (Fig. 2). The VZV channel only detected VZV antigen, not MPXV or other poxviruses, demonstrating the differentiation between OPXV with VZV. The NVAR-OPXV assay detected 10 orthopoxvirus isolates namely AKMV, CPXV-FIN2000, cowpox virus Brighton Red (CPXV-BR), ectromelia virus (ECTV), MPXV clades Ia, Ib, IIa, and IIb, VACV Modified Vaccinia Ankara (VACV-MVA), and VACV Western Reserve (VACV-WR) (Table 4). To further evaluate the obtained results, we aligned the NVAR-OPXV primers and probe with the orthopoxviral DNA sequences tested (Fig. 3). Notably, the sequences within this specific region were found to be identical for MPXV, VACV-WR, VACV-MVA, CPXV-BR and CPXV-FIN2000. CPXV-GER98 and taterapox virus exhibited two base differences in the probe target region, which likely explains its inability to be detected compared to the other two CPXV strains. Variola virus (VARV) displayed three nucleotide mismatches within the probe target region, rendering it undetectable. Borealpox (BRPV), skunkpox, and raccoonpox viruses each exhibited multiple mismatches either in the probe target region or primer binding region and were not detected. No significant matches were found between the probe and the genomes of volepox, deerpox, and batpox viruses. Thus, the MPXV assay successfully detected various MPXV clades, including each of clade Ia, Ib, IIa, and IIb, and demonstrated a high degree of specificity as it targeted MPXV.

## Discussion

In this study, we evaluated a multiplexed real-time PCR on a portable platform (T-COR 8) for the detection of MPXV and differentiation from VZV using clinical specimens and a panel of poxviruses and a commercial VZV antigen/lysate. A total of 116 clinical specimens were tested in the T-COR 8 MPXV/NVAR-OPXV assay with a overall agreement of 99.1% (95% CI, 95.3–100) with the CDC Clade II MPXV real-time PCR assay. For the T-COR 8 MPXV/NVAR-OPXV/VZV assay, 20 MPXV and 19 VZV clinical specimens were analyzed; all results were consistent with prior PCR testing. Notably, one specimen previously only confirmed as negative for Clade II MPXV was identified VZV-positive in this testing. In the specificity evaluation using laboratory grown isolates, the MPXV assay detected all four MPXV clades, Ia, Ib, IIa, and IIb, and AKMV. AKMV was previously observed to cross-react with MPXV PCR, but has only been detected in the country of Georgia and hence may have a limited impact for the current test needs in endemic regions [20]. The VZV assay was reactive only with VZV antigen, supporting the target-specific differentiation within the multiplex assay. A key benefit of this system is that the T-COR 8 thermocycler utilizes crude specimens directly from patients, eliminating the need for nucleic acid extraction. This is particularly advantageous as nucleic acid extraction can be expensive, time-consuming, and extraction kits are one of the most limited resources during outbreaks. Previous results demonstrate that the real-time PCR on crude specimens without extraction is feasible with optimized buffer and dilutions to achieve optimal sensitivity [21]. Additionally, we observe that point-of-care testing with the single-use, gasketed and closable C2T cartridge would eliminate downstream logistics and biosafety concerns, including the need for transport management, inactivation, and additional personal protective equipment (PPE) required for separate laboratory testing.

The portable T-COR 8 thermocycler occasionally produced “invalid” results during the pilot and blind study. Specifically, 3 out of 88 samples (3.5%) in the pilot study and 2 out of 28 samples (7.1%) in the blind study resulted in invalid result. As per the protocol from Tetracore, any samples yielding an invalid signal were subjected to re-testing. During testing we did not observe that the occurrence of an invalid signal resulted from any technical or sample preparation issues. Rather, it indicated a failure to detect sufficient amplification, rendering it incapable of providing interpretations. Upon re-testing, each clinical sample yielded the expected results. Additionally, there was one false positive sample in the pilot study. This sample had a Ct value of 20.1 when tested with the T-COR 8 thermocycler; upon retesting of this one sample from the same aliquot, a negative result was obtained. We suspect that the inconsistent result may have been caused by sample handling error or mis-pipetting, but cannot rule out a potential cartridge-related artifact. Excluding initial invalids and the probable pipetting error, results found from clinical remainder specimens had 100% concordance with laboratory-based testing.

In addition to qualitative result concordance (positive vs. negative), we also compared the T-COR 8 MPXV Ct values the laboratory test to examine if there were any unexpected findings. We note that the MPXV clade II laboratory test utilized 4 µL of swab eluate (pre-extraction) compared to 12.5 µL of eluate for the T-COR 8. If extraction retained all viral DNA and the reaction efficiencies were equivalent, this volume differential would on average lead to a higher Ct (+ 2.5 Ct) with the laboratory-based test given lower input quantity of target nucleic acid. Here we found on average, across all correctly identified positive samples, a lower Ct (-0.6 Ct) with the lab test (mean Ct = 27.9) relative to T-COR 8 (mean Ct = 28.4), indicating some reduced efficiency of the T-COR 8 test. These results align with data from a separate assessment of the instrument dynamic range (supplemental Fig. 2), where the lowest genome copy equivalents detected by the TCOR was 16 copies/reaction using synthetic DNA, relative to previously reported sensitivity to extracted genome DNA for NVAR-OPXV (~ 2.5 fg DNA, or 12.5 genome copies) [12]. Among Ct differences (Ct∆) between the prior lab-based real-time PCR and new T-COR 8 MPXV results, analyses by patient sex, age, or days post rash (DPR) onset for sample collection, only DPR achieved significance. However, interpretation of this finding is limited by the substantial proportion of missing metadata, particular for DPR, as well as the small sample size within subgroups. One possible explanation is that the laboratory-based extraction process is less efficient with samples collected later during the course of illness, or alternatively that there may be reduced inhibitors present as lesions begin to scab over and heal, with subsequent improvement in the T-COR 8 extraction-free testing. However, interpretation of this finding is limited by the substantial proportion of missing metadata, particular for DPR, as well as the small sample size within subgroups.

Despite the insights gained from this study, several limitations should be acknowledged. The clinical specimens and laboratory cultured virus isolates were not standardized for genome equivalents or nucleic acid concentrations. As a result, variability in input material may have influenced detection outcomes, particularly in the specificity analysis. The failure of detection may reflect low input material rather than true absence of the target. Second, the MPXV primers in MPXV/NVAR-OPXV/VZV assay used were generic MPXV primers and were not designed to exclude AKMV. This results in the detection of both MPXV and AKMV in MPXV assay channel. This represents a limitation in analytical specificity, however, AKMV has been reported only in limited geographic regions and is unlikely to significantly impact detection in current endemic outbreak settings. Additionally, the clinical sample size in this study was limited, and all evaluations were conducted in a controlled laboratory setting rather than under true field conditions. Therefore, further testing are needed to evaluate assay performance in real-world and resource-limited environments.

In summary, the multiplex T-COR 8 assays demonstrated consistent detection of MPXV and VZV in clinical samples and accurately distinguished between the two viruses. Specificity evaluation across 19 poxvirus isolates and a commercial VZV antigen/lysate showed that the MPXV assay detected MPXV and AKMV, whereas the VZV assay was reactive only with VZV strains, supporting the target-specific differentiation within the multiplex assay. Overall, these findings support the analytical reliability of the T-COR 8 assay under laboratory evaluation conditions and its potential for further development and validation in expanded diagnostic settings.

## Supplementary Information


Supplementary Material 1.



Supplementary Material 2.


## Data Availability

The datasets used and analyzed during the current study are available from the corresponding author on reasonable request.

## Footnotes

1
^§^See e.g., 45 C.F.R. part 46; 21 C.F.R. part 56; 42 U.S.C. § 241(d), 5 U.S.C. § 552a, 44 U.S.C. § 3501 et seq.
