# Supplementary material for: Evaluation of a portable multiplex real-time pcr assay for the detection of monkeypox virus and non-variola orthopoxviruses
Source: Virol J. 2026 Jun 22;23:206. doi: 10.1186/s12985-026-03195-1 (PMC13536642; doi:10.1186/s12985-026-03195-1)
Supplement: Supplementary file 1 [file 12985_2026_3195_MOESM1_ESM.docx]

Supplemental Table 1: Viruses utilized in this study

| **Virus Name** | **Abbreviation** | **GenBank ID** |
| --- | --- | --- |
| Akhmeta virus | AKMV | MH607141 |
| Batpox virus | Batpox | PP711852 |
| Borealpox virus | BRPV | MN240300 |
| Cowpox virus_Germany_1998_2 | CPXV-GER98 | HQ420897 |
| Cowpox virus_Brighton Red | CPXV-BR | AF482758 |
| Cowpox virus_Finland2000_Man | CPXV-Fin2000 | HQ420893 |
| Deerpox virus | Deerpox | MF966153 |
| Ectromelia virus | ECTV | NC_004105 |
| Monkeypox virus_ (Clade Ia) | MPXV Ia | n/a |
| Monkeypox virus_ (Clade Ib) | MPXV Ib | n/a |
| Monkeypox virus_USA_2003_44 | MPXV IIa | DQ011153 |
| Monkeypox virus_ USA_2022_MA001 | MPXV IIb | ON563414 |
| Raccoonpox virus | Raccoonpox | NC_027213 |
| Skunkpox virus | Skunkpox | NC_031038 |
| Taterapox virus | TATV | NC_008291 |
| Vaccinia virus_Modified Vaccinia Ankara | VACV-MVA | AY603355 |
| Vaccinia virus_Western Reserve | VACV-WR | AY243312 |
| Variola virus_V68-258 | VARV-Banu | DQ437581 |
| Volepox virus | Volepox | NC_031033 |

Supplemental Table 2: Pilot study samples and test results

Supplemental Table 3: Blind study samples and results

Supplemental Figure 1. Alignment of NVAR-OPXV (E9L) primers and probe with orthopoxviral DNA. The primers and probe for each are aligned with the targeted sequence of orthopoxviral species. E9L target gene is presented as found in the genome on the reverse strand. Volepox, deerpox, batpox, tanapox are not shown (6+ mismatches on any target sequence). MPXV clades Ia, Ib, and IIa are not included. Primer sequences are Forward (TCAACTGAAAAGGCCATCTATGA) Probe (CCATGCAATATACGTACAAGATAGTAGCCAAC), and Reverse (GAGTATAGAGCACTATTTCTAAATCCCA).

Supplemental Figure2. Differences observed in MPXV positive sample Ct values between the lab-based Clade II (extraction) and T-COR 8 (extraction free) testing based on (A) age and (B) days post rash/symptom onset. The presumed false positive test, inconclusive, or invalid results were excluded from analysis. No significant difference was observed between groups based on age. Samples collected at 11+ days had a higher Ct in lab based Clade II testing than on the T-COR 8.

Supplemental Figure 3. Analytic Sensitivity assessment using synthetic DNA on the T-COR 8. Synthetic DNA containing Armored DNA Quant ™ MPOX/Non-Variola Orthopox (Bio-techne, Minneapolis, USA) was serially diluted and tested using the MPXV/NVAR-OPXV assay and resulsting Ct Values. The lowest detected genome equivalents was 16 copies (Ct 34.6) for MPXV assay and 50 copies (Ct 34.1) for NVAR-OPXV assay. Data shared by Tetracore, Inc.
