# Supplementary material for: Evaluation of a portable multiplex real-time pcr assay for the detection of monkeypox virus and non-variola orthopoxviruses
Source: Virol J. 2026 Jun 22;23:206. doi: 10.1186/s12985-026-03195-1 (PMC13536642; doi:10.1186/s12985-026-03195-1)
Supplement: Supplementary file 2 [file 12985_2026_3195_MOESM2_ESM.docx]

**Supplemental Table 1: Viruses utilized in this study**

| **Virus Name** | **Abbreviation** | **GenBank ID** |
| --- | --- | --- |
| Akhmeta virus | AKMV | MH607141 |
| Batpox virus | Batpox | PP711852 |
| Borealpox virus | BRPV | MN240300 |
| Cowpox virus_Germany_1998_2 | CPXV-GER98 | HQ420897 |
| Cowpox virus_Brighton Red | CPXV-BR | AF482758 |
| Cowpox virus_Finland2000_Man | CPXV-Fin2000 | HQ420893 |
| Deerpox virus | Deerpox | MF966153 |
| Ectromelia virus | ECTV | NC_004105 |
| Monkeypox virus_ (Clade Ia) | MPXV Ia | n/a |
| Monkeypox virus_ (Clade Ib) | MPXV Ib | n/a |
| Monkeypox virus_USA_2003_44 | MPXV IIa | DQ011153 |
| Monkeypox virus_ USA_2022_MA001 | MPXV IIb | ON563414 |
| Skunkpox virus | Skunkpox | NC_031038 |
| Taterapox virus | TATV | NC_008291 |
| Vaccinia virus_Modified Vaccinia Ankara | VACV-MVA | AY603355 |
| Vaccinia virus_Western Reserve | VACV-WR | AY243312 |
| Raccoonpox virus | Raccoonpox | NC_027213 |
| Variola virus_V68-258 | VARV-Banu | DQ437581 |
| Volepox virus | Volepox | NC_031033 |

**Supplemental Table 2 Primers and probe information^a,b,c^**

| **MPXV-F-20** | Monkeypox Virus Forward Primer | 5’-GGA AAA TGT AAA GAC AAC GAA TAC AG-3’ |
| --- | --- | --- |
| **MPXV-R-20** | Monkeypox Virus Reverse Primer | 5’-GCT ATC ACA TAA TCT GGA AGC GTA-3’ |
| **MPXV-P-5** | Monkeypox Virus Probe | 5’-FAM-AAG CCG TAA TCT A(T-BHQ1) GT TGT CTA TCG TGT CC-Spacer C6-3’ |
| **NVAR-OPXV-F-20** | Non-Variola Orthopoxvirus Forward Primer | 5’-TCA ACT GAA AAG GCC ATC TAT GA-3’ |
| **NVAR-OPXV-R-20** | Non-Variola Orthopoxvirus Reverse Primer | 5’-GAG TAT AGA GCA CTA TTT CTA AAT CCC A-3’ |
| **NVAR-OPXV-P-5** | Non-Variola Orthopoxvirus Probe | 5’-Cal Fluor Red-CCA TGC AAT A (T–BHQ1) A CGT ACA AGA TAG TAG CCA AC-Phos-3’ |
| **VZV-FOR** | VZV Forward primer | 5’-GCT CTC TTT CTG TGT GTC CAC-3’ |
| **VZV-REV** | VZV Reverse primer | 5’-TCG ACT GGC TGG GAC TT-3’ |
| **VZV-PROBE** | VZV Probe | 5’-ATTO647NN/ATC GTT TAC TAO GAA CTC CGC GCA A -3’ |

^a^Tetracore assay for MPXV DNA utilizes two forward and two reverse primers targeting intergenic region between OPG205 and OPG208 and a FAM labeled probe.

^b^Tetracore IC is labeled with ATTO 550 (read in the DFO channel) and it is an exogenous IC which is included in the reaction mix to monitor inhibition.

^c^RNase P primers and probe labeled with ATTO 647 (read in the Cy 5 channel) are also added in the premixed reagents for monitoring the amplification as an endogenous IC.

Supplemental Table 3: Pilot study samples and test results

Supplemental Table 4: Blind study samples and results

Supplemental Figure 1. Differences (Ct ∆) observed in MPXV positive sample Ct values between the lab-based Clade II (extraction) and T-COR 8 (extraction free) MPXV testing based on (A) age and (B) days post rash/symptom onset. The presumed false positive test, Clade II inconclusive (Ct between 37 and 40) and negatives (Ct >40), and invalid results were excluded from analysis. No significant difference was observed between groups based on age. Samples collected at 11+ DPR had a higher Ct in lab based Clade II testing than on the T-COR 8. Lines indicate Mean and Standard Deviation. P-values were calculated using Tukey’s comparison.

Supplemental Figure 2. Analytic Sensitivity assessment using synthetic DNA on the T-COR 8. Synthetic DNA containing Armored DNA Quant ™ MPOX/Non-Variola Orthopox (Bio-techne, Minneapolis, USA) was serially diluted and tested using the MPXV/NVAR-OPXV assay and resulsting Ct Values. The lowest detected genome equivalents was 16 copies (Ct 34.6) for MPXV assay and 50 copies (Ct 34.1) for NVAR-OPXV assay. Data shared by Tetracore, Inc.
